# Long Term Storage of Dry versus Frozen RNA for Next Generation Molecular Studies

**DOI:** 10.1371/journal.pone.0111827

**Published:** 2014-11-07

**Authors:** Eric Seelenfreund, William A. Robinson, Carol M. Amato, Aik-Choon Tan, Jihye Kim, Steven E. Robinson

**Affiliations:** Department of Medical Oncology, University of Colorado Denver, Anschutz Medical Campus, Aurora, Colorado, United States of America; U.S. Geological Survey, United States of America

## Abstract

The standard method for the storage and preservation of RNA has been at ultra-low temperatures. However, reliance on liquid nitrogen and freezers for storage of RNA has multiple downsides. Recently new techniques have been developed for storing RNA at room temperature utilizing desiccation and are reported to be an effective alternative for preserving RNA integrity. In this study we compared frozen RNA samples stored for up to one year to those which had been desiccated using RNAstable (Biomatrica, Inc., San Diego, CA) and stored at room temperature. RNA samples were placed in aliquots and stored after desiccation or frozen (at −80°C), and were analyzed for RNA Integrity Number (RIN), and by qPCR, and RNA sequencing. Our study shows that RNAstable is able to preserve desiccated RNA samples at room temperature for up to one year, and that RNA preserved by desiccation is comparable to cryopreserved RNA for downstream analyses including real-time-PCR and RNA sequencing.

## Introduction

In order to perform genomic research, nucleic acids must first be isolated, purified, and stored before downstream analyses can be performed. DNA is a very stable molecule and preserves well, conversely RNA is highly labile and degrades quickly if stored in improper conditions. Aqueous RNA can be degraded by spontaneous phosphodiester bond cleavage as a result of acid or base catalyzed transesterification from the intramolecular nucleophilic attack of the 2′ hydroxyl group on the phosphorous atom [1]. Additionally, ribonucleases (RNases) which enzymatically degrade aqueous RNA are nearly ubiquitous in all cells and pose a constant threat of contamination and degradation of purified RNA. Traditionally RNA is stored at −20°C, −80°C or in liquid nitrogen to provide protection from these degradative reactions. While storage and shipment of nucleic acids in freezers can be effective and sufficient for maintaining high quality samples, power supplies and freezers themselves are not failsafe. Shipping frozen RNA on dry ice is expensive, requires special handling and is subject to air-travel regulations and is time sensitive. To exemplify the inherent problems of relying on freezers, recently millions of dollars of bio-specimens have been lost as a result of power and freezer failures during Hurricane Sandy [2] and at a Harvard-associated hospital due to an alarm failure [3]. Additionally, alternative methods of storing nucleic acids should be considered on the grounds that Ultra-Low-Temperature (ULT) freezers, which can cost up to $20,000, run continuously for years, take up large areas of space, and require tremendous amounts of energy. Government estimates report that these freezers require 20–70 kWh of energy per day to operate and can each generate up to 35,000 pounds of carbon dioxide per year [4].

For years researchers have been investigating more effective means of storing and shipping RNA [5], [6], [7], [8]. We evaluated the efficacy of desiccating and storing RNA for use in molecular studies. For RNA desiccation we used RNAstable, a novel storage medium produced by Biomatrica, which mimics the natural mechanisms of anhydrobiosis (life without water) which has evolved in various small multicellular organisms including tardigrades and brine shrimp [9]. Tardigrades, colloquially known as water bears, for example, can survive in a desiccated state for at least 120 years until being rehydrated [10]. While long term whole-organism survival requires many specific adaptations, cells with all of their biochemical components can be desiccated and rehydrated without functional loss with the mere addition of trehalose or other disaccharides [11]. RNAstable reportedly acts as trehalose does within anhydrobiotic organisms and can form a “glass-like shell” around a desiccated RNA sample [9], protecting the nucleic acid from the ubiquitous RNases and subsequent degradation: therefore making it ideal for the storage and transport at ambient temperatures.

Research studies have examined the efficacy of the RNAstable system for storing desiccated RNA at room temperature, but these mainly focused on short term storage. RNAstable has been shown to be effective for preserving and maintaining desiccated viral RNA levels for up to 92 days as assayed by real time PCR [5] and for preserving desiccated RNA of sufficiently high quality for downstream microarray analysis for up to five weeks [6]. RNAstable has also been shown to preserve ribosomal and messenger RNA for the short period of time that RNA may be in transit during shipping, and that after a period not exceeding two weeks, the desiccated RNA can be rehydrated without losing any RNA yield [8]. Biomatrica has reported that RNA stored using RNAstable after 29 months is suitable for qPCR and displays high RNA Integrity Numbers (RIN); however, they are only analyzing ribosomal RNA (http://www.biomatrica.com/rnastable.php). Our study aims to test the effectiveness of RNAstable of preserving high quality RNA for long term storage, and most importantly, focus on the preservation of messenger RNA and the suitability of desiccated RNA for RNA sequencing.

To study the effectiveness of RNAstable's ability to preserve RNA at room temperature, we performed RNA integrity analysis, quantitative RT-PCR, and next generation RNA sequencing with subsequent bioinformatics analysis. RNA Integrity Number (RIN) has been traditionally used as a measure of RNA quality, but a large part of the RIN calculation stems from ribosomal RNA integrity, therefore RIN represents an incomplete analysis of RNA degradation and quantification of message RNA in a specimen destined for gene expression analysis. RIN score has, for example, been found to be uncorrelated with the PCR efficiency of RNA samples undergoing qPCR [12]. Therefore, to get a meaningful comparison of the RNA quality of our stored samples, in addition to performing RIN analysis, we compared desiccated and frozen samples and their message levels by qPCR expression of *TBP* and compared the results of next generation whole transcriptome RNA sequencing (RNA-Seq).

RNA-Seq is a novel genomic sequencing technique that allows for thorough mapping and quantitating of the transcriptome. RNA-Seq allows one to quantitatively analyze a whole organism's transcriptome, and to analyze novel sequences, transcript isoforms, and all sizes and types of non-coding RNAs to a single base resolution [13]. Next generation sequencing techniques including RNA-Seq will become ubiquitous in future genomics research; therefore desiccation would not be a very useful storage technique unless it was shown to maintain RNA of sufficiently high quality for effective RNA sequencing.

## Results

### Integrity of stored RNA

**Figure 1:**
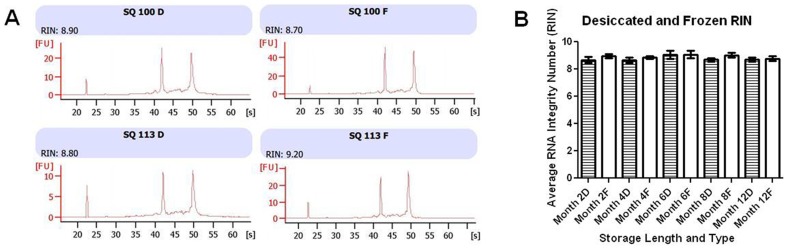
RNA Integrity Number analysis of stored RNA. (**A**) RIN Electrophoretic Analysis of RNA integrity from two representative RNA samples which were desiccated and stored at room temperature (D) or Frozen at −80°C (F) for 12 months. Desiccated samples are shown on the left and frozen samples are shown on the right. The two main peaks in each electropherogram are representative of 18S and 28S ribosomal RNA fragments. (**B**) The average RIN values with standard error of the mean from all RNA samples measured after the given number of months in storage in a desiccated (D) or frozen (F) state.

We analyzed the integrity of the stored RNA samples by Agilent 2100 RIN number. Paired frozen and desiccated aliquots of total human RNA samples were analyzed every two months (except at 10 months) for one year of total storage. The integrity of RNA samples that were frozen at −80°C remained consistent, with average RIN scores ranging from 8.8 to 9.1. The integrity of RNA samples that were desiccated and then stored at room temperature were found to maintain average RIN values of between 8.7 and 9.1 at the five tested time points (**Fig. 1A, B**). The largest discrepancy in RIN value between two paired samples was 0.8 with the SQ 146 desiccated RNA sample having a 0.8 lower RIN score then its frozen counterpart at month two and month four of storage. The average difference between paired desiccated and frozen sample RIN values was 0.2 with frozen samples having the slightly higher average RIN values (**Fig. 1B**).

### RT-qPCR validation of long-term desiccated RNA

**Figure 2:**
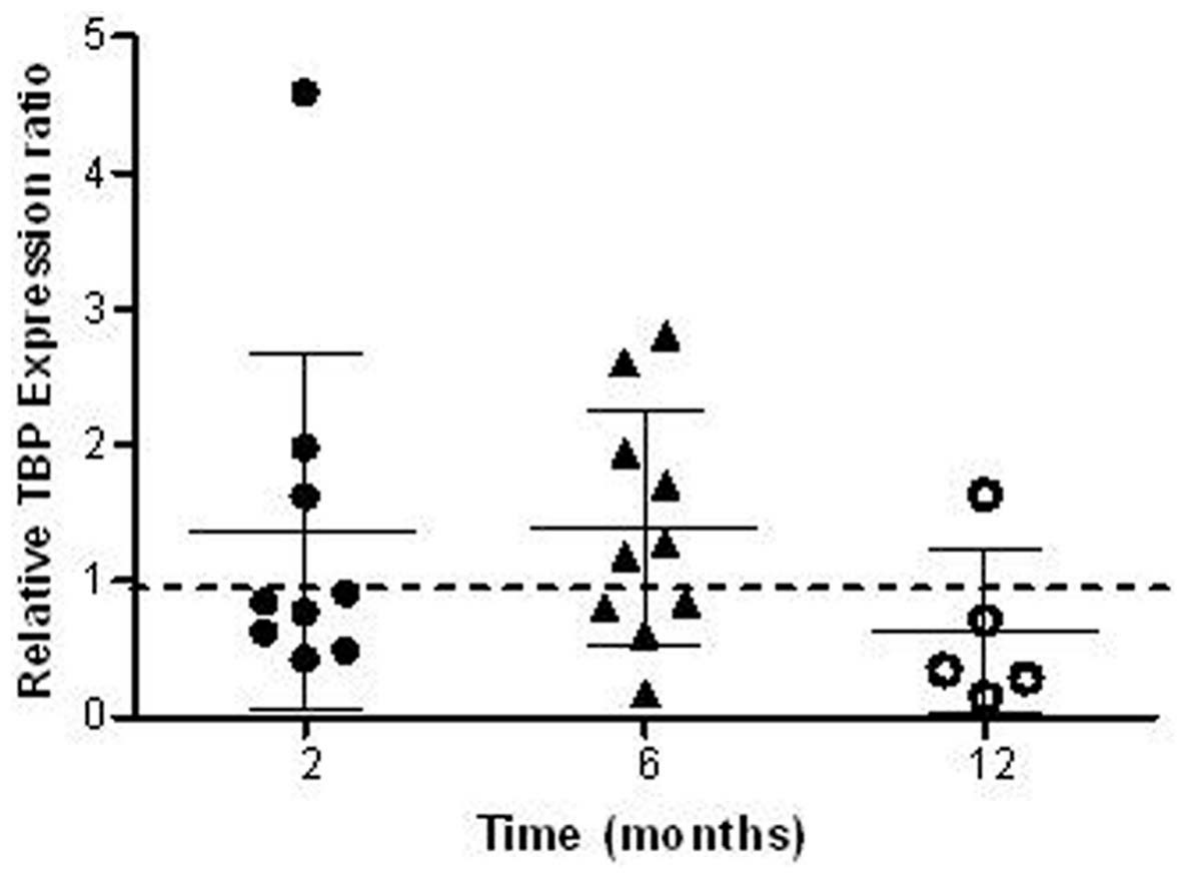
Preservation of *TBP* mRNA in desiccated versus frozen samples. Stored RNA samples are analyzed after two, six and 12 months by RT-qPCR for one year. The values depicted are *TBP* expression levels in each desiccated sample relative to its matched frozen aliquot. Error bars represent standard error of the mean among the paired ratios in each time point.

After two, six and 12 months of storage, up to 10 patient RNA samples were analyzed from both frozen and desiccated aliquots. At the two and six month time points the majority of the tested desiccated samples expressed *TBP* at levels close to or exceeding the expression of *TBP* from the source-matched frozen RNA aliquot. Desiccated-to-frozen *TBP* expression ratios of one indicate equivalent expression of *TBP* in the frozen and desiccated aliquots, and ratios above one indicate that there are higher *TBP* message levels in the desiccated aliquots. The average of the relative *TBP* expression ratios of the paired desiccated to frozen samples was found to be above one after two and six months of storage. After one year of storage, five paired desiccated and frozen RNA samples were analyzed and only one RNA source had a *TBP* desiccated-to-frozen expression ratio above one but the average of these five ratios was still within half a standard error of the mean to a value of one (**Fig. 2**).

Over the yearlong study the C_t_ values for qPCR *TBP* amplification generally increased across all samples as storage time increased. Average C_t_ values from desiccated and frozen aliquots of five samples at each time point (four at the two month time point) are shown in **Table S1**. All RNA samples, whether frozen or desiccated, were found to have a higher C_t_ value after 12 months of storage compared to after two months of storage. From the two month checkpoint to the year checkpoint the C_t_ value of the desiccated RNA samples increased on average by 2.85 cycles whereas the corresponding frozen samples showed an increase of 1.85 cycles (**Table S1**). The trend of increasing C_t_ number did not hold for each sample, as fluctuating values were seen across the RNA specimens over the yearlong study. All C_t_ values from frozen and desiccated samples stayed within the range of the upper 20 s to mid-30 s in cycle numbers representing positive *TBP* expression.

qPCR validation experiments were then conducted using three additional primer/probe sets from the Roche Applied Sciences Universal ProbeLibrary Set. cDNA from RNA which was stored desiccated or frozen for 12 months was analyzed for relative expression of *ACTB, GAPD* and *GUSB* message in addition to *TBP*. Relative desiccated-to-frozen RNA expression level ratio averages were very consistent across the four genes after a year of storage (**Fig. S1**), and high and low ratio outliers were also consistent across the four genes tested.

### RNA sequencing analysis of stored RNA

**Figure 3:**
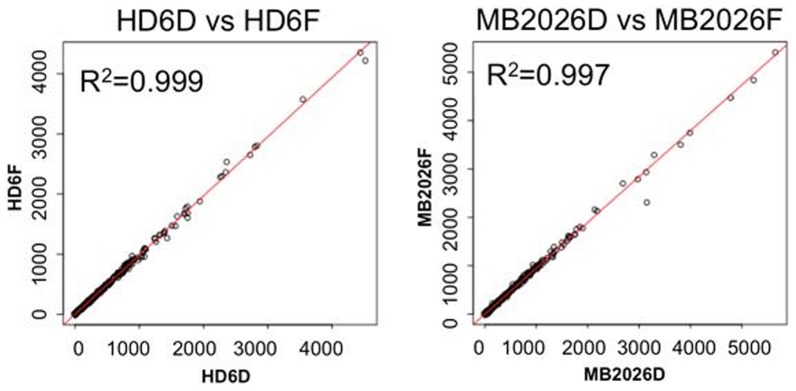
Gene expression comparisons between the RNA stored by desiccated and frozen methods for 76 days. The left panel illustrates the comparison of RNA from HD6D (desiccated aliquot) and HD6F (frozen aliquot). The right panel illustrates the comparison of RNA from MB2026D (desiccated aliquot) and MB2026F (frozen aliquot). X- and Y-axis represent the gene expression values in FPKM values.

Total RNA was extracted from two human blood samples. Each sample was analyzed by RNA-Seq after no storage (HD6, MB2026), after 76 days of storage desiccated at room temperature (HD6D, MB2026D) or after being frozen at −80°C (HD6F, MB2026F). Nearly 97% of all sequence tags from fresh, frozen and desiccated RNA aliquots of both samples could be mapped to HG19 (**Table 1**). **Figure 3** illustrates the pair-wise gene expression comparison plots for the different storage methods in these two samples. From these gene expression comparisons, RNA extracted from fresh samples (HD6 and MB2026) and compared to the desiccated (HD6D, MB2026D) or frozen (HD6F and MB2026F) shared high degree of correlations (R^2^ = 0.93) (**Fig. S2**). Furthermore, the gene expressions obtained from desiccated (HD6D, MB2026D) and frozen (HD6F and MB2026F) methods are nearly identical (R^2^ = 0.997 and 0.999) (**Fig. 3**). This indicates that the RNA stored in a desiccated state maintained the same gene expression profiles as the RNA stored in the traditional frozen method.

**Table 1:** RNA-Seq statistics of sequence tags mapped to the human genome (HG19) from fresh RNA or RNA stored frozen or desiccated for 76 days from the Skin Cancer Biorepository samples HD6 and MB2026.

Sample		Fresh	Desiccated	Frozen
	# Sequence tags	66,830,230	46,816,482	50,129,753
HD6	# Sequence tags mapped to HG19	64,819,678	45,310,783	48,404,953
	% mapped	97	96.8	96.6
	# Sequence tags	84,076,804	49,406,447	39,417,648
MB2026	# Sequence tags mapped to HG19	81,223,581	47,865,052	38,142,137
	% mapped	96.6	96.9	96.8

## Discussion

### mRNA is preserved by desiccated room temperature storage

Total extracted RNA which had been desiccated and stored for up to one year at room temperature was found to maintain very high overall integrity. RIN analysis showed that total RNA can remain stable for one year of desiccated storage and that desiccation is comparable to −80°C frozen storage; the industry standard storage method for preserving total RNA integrity. Desiccating total RNA also preserves mRNA for downstream reverse transcription and qPCR analysis. RNA which was stored for up to one year was found to contain a sufficient amount of high quality mRNA transcripts of the genes tested to allow for qPCR detection after rehydration and reverse transcription. In fact, after six months of storage, when analyzed by qPCR, threshold cycles for *TBP* from desiccated RNA samples were lower on average than the C_t_ values from the same RNA which had been stored frozen at −80°C, indicating that desiccation can even be superior to −80°C frozen storage in terms of preserving RNA for downstream analysis.

After 12 months of storage the desiccated-to-frozen RNA gene expression ratios dropped relative to the two and six month checkpoints, though the differences between these ratios are not statistically significant. qPCR carries with it an inherent variability, which may explain the differences seen. Small pipetting errors can result in highly varied C_t_ values after exponential DNA amplification. Running samples in triplicate and averaging the results helps to reduce possible errors but qPCR should not be relied on completely. Individual samples varied in calculated *TBP* C_t_ value across the time points (**Table S1**) in ways that would not be predicted from mere difference in storage length. C_t_ numbers would not be expected to go down after an RNA sample is stored for more time, but for some of the samples tested, C_t_ numbers dropped from month two to month six or from month six to month 12. The fact that the measured desiccated-to-frozen gene expression level ratios as measured by qPCR were lower at the 12 month time point could well be accounted for by the qPCR C_t_ variability. Regardless of any possible errors or variability, C_t_ values for *TBP* expression remained in the low to mid 30 s throughout the yearlong study for both desiccated and frozen samples representing preservation of mRNA.

### RNA sequencing of desiccated and frozen RNA results in near identical gene expression profiles

After being stored for 76 days at either room temperature in a desiccated state or at −80°C, RNA aliquots of two whole RNA extracts stored at these conditions were compared by RNA sequencing on gene expression of 14,114 genes. Transcriptome-wide expression profiles from RNA samples which were stored desiccated or frozen were very similar, indicating that the RNA stored by the desiccated method maintained the same gene expression profiles as the RNA stored in the traditional manner.

In summary, desiccation appears to be a very promising technique for long term RNA storage. Storage in a desiccated state preserves RNA integrity and allows for robust downstream standard and next-generation transcriptomic analysis, and as such should be seriously considered in any modern biotechnical institute that deals with RNA. As desiccated room temperature storage has been shown to be effective at preserving high quality RNA, desiccation of RNA may also one day supplant ULT freezers for RNA storage, and would thus reduce laboratory reliance upon carbon-based power grids. Therefore, use of RNA desiccation techniques may save research groups energy costs, while simultaneously reducing their carbon footprint and significantly allaying the present fears associated with the real possibility of freezer failures.

## Materials and Methods

### Sample collection and ethics statement

Blood was collected by the University of Colorado Skin Cancer Biorepository. Peripheral blood was collected in PAXgene RNA blood tubes (Qiagen/Becton Dickson) from 12 patients in the Cutaneous Oncology Clinic at the University of Colorado Hospital (UCH). Informed written consent was obtained from all subjects and this study was approved by the Colorado Multiple Institutional Review Board (COMIRB) No: 05-0309 at the University of Colorado Denver.

### RNA processing and storage

Total RNA was extracted from PAXgene RNA blood tubes using the PAXgene Blood RNA Kit (PreAnalytix, Hilden, Germany) according to the manufacturer's protocol using only RNase-free equipment and solutions. The RNA was eluted in 80 µl of nuclease free H_2_O. RNA yield was quantified using a NanoDrop 1000 spectrophotometer. Each isolated RNA sample was divided into 12 aliquots of 6 µl each, half of which were pipetted into 1.5 ml Eppendorf tubes and immediately stored at −80°C. The remaining six samples isolated from each blood sample were desiccated in Biomatrica RNAstable 1.5 ml tubes by room temperature vacuum centrifugation using an Eppendorf Speed Vacuum Concentrator (Eppendorf, Hamburg, Germany). The desiccated RNA aliquots were then stored in a special humidity controlled dry storage cabinet. Paired aliquots of each sample (frozen and desiccated) were analyzed every two months (except at 10 months), RNAstable tubes were rehydrated in 6 µl of nuclease-free H_2_O, and the RNA quality was analyzed using an Agilent 2100 bioanalyzer to obtain RINs and by quantitative real time PCR at two, six, and 12 months (**Fig. S3**).

### cDNA Synthesis

cDNA was synthesized from 100 ng of total RNA from both frozen and rehydrated samples using the Roche Transcriptor First Strand cDNA Synthesis kit according to the manufacturer's instructions.

### Quantitative real time-PCR

Message levels of the housekeeping genes TATA-Binding Protein (*TBP*), Beta-actin (*ACTB*), Glyceraldehyde-3-Phosphate Dehydrogenase (*GAPD*), and beta-glucuronidase (*GUSB*) were assessed by qPCR. Primer and probe mixes were obtained from Roche Applied Science as part of the Universal ProbeLibrary Set, Human Reference Gene Assays. For the real-time-PCR assays, 20 µl reactions were run, each in triplicate. The PCR conditions were set for the UPL Roche designed protocol of two minutes at 50°C then 10 minutes at 95°C then 44 cycles of denaturation at 95°C, primer annealing at 60°C and polymerization at 72°C for ten, thirty, and one seconds respectively. qPCR reactions were performed on a BIORAD CFX 96 Real-Time System c1000 Thermal Cycler. A standard threshold of 32 RFU was set for comparison of RNA samples run on different plates, and RNA levels were compared pairwise as delta C_t_ ratios of desiccated to frozen samples.

Frozen RNA was thawed and desiccated RNA was rehydrated after two, six, and 12 months and was reverse transcribed into cDNA. RT-qPCR was performed and the expression of *TBP* from desiccated RNA relative to frozen RNA was calculated using the following formula: 2^−(D-F)^.

For D being the average of the triplicate threshold cycle C_t_ values of a given desiccated RNA sample and F being the average of the triplicate threshold cycle C_t_ values from the frozen RNA aliquot of the same sample.

### RNA Integrity Number

The RNA Integrity or RIN value was calculated using the Agilent 2100 Bioanalyzer with RNA Nano chips at the University of Colorado Denver Genomics and Microarray Core.

### RNA-Seq

Total RNA was extracted from blood samples HD6 and MB2026 stored in the University of Colorado Skin Cancer Biorepository. Both RNA samples were eluted twice with 20 µl nuclease free H_2_O. RNA yield was quantified by a NanoDrop 1000 spectrophotometer. Each RNA sample was pipetted into three, 12 µl aliquots. One aliquot from each sample was sent immediately for RNA-Seq, another was placed at −80°C and the last aliquot was desiccated and stored as described above. After 76 days the desiccated samples were rehydrated in 12 µl of nuclease free H_2_O and the frozen aliquots were thawed on ice. These aqueous RNA samples were sent on ice for whole transcriptome sequencing (RNA-Seq) (**Fig. S4**).

Total RNA was extracted and converted into a sequencing library according to the manufacturer's protocol (Illumina Inc., San Diego, CA). These libraries were sequenced on the Illumina HiSeq 2000 sequencer with 1×50 (single-end, 50 bases). On average, 56 million (range: 39–84 million) reads were obtained from each sample. RNA-Seq analysis was performed as previously described [14], [15] using the TopHat/Cufflinks workflow. In brief, sequencing reads were mapped against the human genome using TopHat (version 1.4.1) [16]. We used the University of California, Santa Cruz (UCSC) reference annotation (HG19) as the mapping guide, and allowed two mismatches for the initial alignment and two mismatches per segment with 25 base pair segments. On average, 96.8% (96.9–97%) of the reads aligned to the human genome. Next, we employed Cufflinks (version 1.3.0) [17] to assemble the transcripts using the RefSeq annotation as the guide, but allowed for novel isoform discovery in each sample. Isoforms were ignored if the number of supporting reads were less than thirty and if the isoform fraction was less than 10% of the gene. The data were fragment bias corrected, multi-read corrected, and normalized by the total number of reads. The transcript assemblies for each sample were merged using cuffmerge [18]. We next computed the transcript's fragment per kilo base mapped (FPKM) values by rerunning Cufflinks using the merged assembly as the guide. Gene expression was estimated by summing the FPKM values of multiple transcripts that represent the same gene. Plots and R-squared values were obtained by using R/Bioconductor (R version 2.15.2).

## Supporting Information

Figure S1 **The desiccated to frozen (D:F) ratios of three genes relative to the TBP D:F ratio from five RNA samples.** Box and whisker plots are shown, indicating the spread of the data from the five samples. A value of one indicates that the preservation of RNA in the desiccated sample relative to its frozen aliquot is the same for TBP and the listed gene.
(DOCX)

Figure S2 **Gene expression comparisons between fresh RNA and RNA stored by desiccated and frozen methods for 76 days.** The top panels illustrate the comparison of RNA from HD6D (desiccated aliquot) and HD6F (frozen aliquot) to HD6 (the fresh HD6 RNA aliquot). The bottom panels illustrate the comparison of RNA from MB2026D (desiccated aliquot) and MB2026F (frozen aliquot) to MB2026 (the fresh MB2026 RNA aliquot). X- and Y-axis represent the gene expression values in FPKM values.
(DOCX)

Figure S3 **Flow chart of desiccated-frozen RNA evaluation scheme.** In the diagrams of microcentrifuge tubes, grey indicates aqueous RNA, yellow indicates desiccated RNA, and white indicates frozen RNA.
(DOCX)

Figure S4 **RNA-Seq sample preparation flowchart.** In the diagrams of microcentrifuge tubes, grey indicates aqueous RNA, yellow indicates desiccated RNA, and white indicates frozen RNA.
(DOCX)

Table S1 **qPCR threshold cycle (C_t_) values for five frozen and desiccated samples using Roche TBP primer/probe mixes after two, six, and 12 months of storage.**
(DOCX)
