# Supplementary figures and images for: Long Term Storage of Dry versus Frozen RNA for Next Generation Molecular Studies
Source: PLoS One. 2014 Nov 7;9(11):e111827. doi: 10.1371/journal.pone.0111827 (PMC4224406; doi:10.1371/journal.pone.0111827)

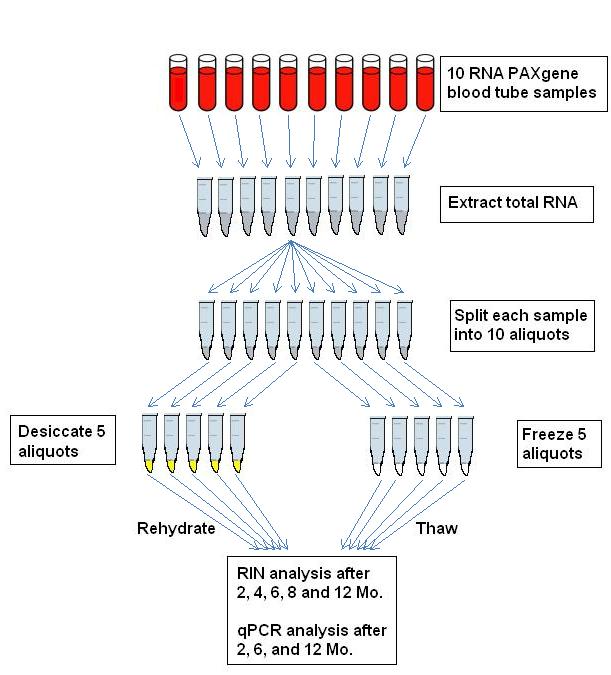

Supplement: Figure S3 — Flow chart of desiccated-frozen RNA evaluation scheme. In the diagrams of microcentrifuge tubes, grey indicates aqueous RNA, yellow indicates desiccated RNA, and white indicates frozen RNA. (DOCX) [file pone.0111827.s003.docx]

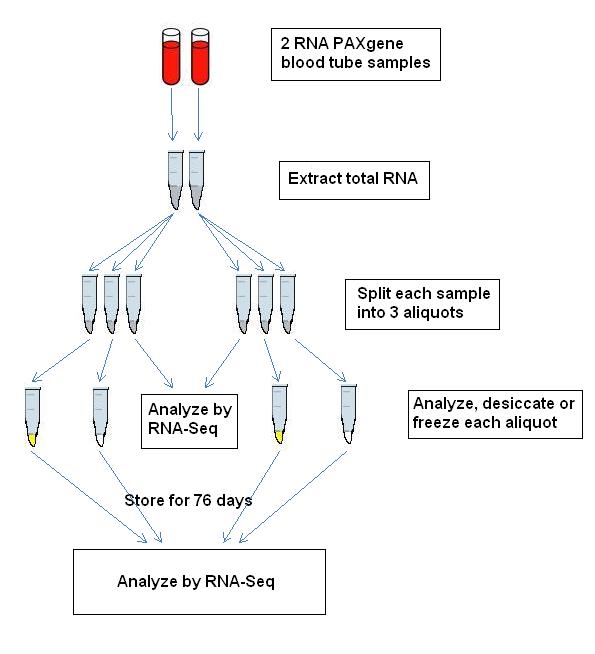

Supplement: Figure S4 — RNA-Seq sample preparation flowchart. In the diagrams of microcentrifuge tubes, grey indicates aqueous RNA, yellow indicates desiccated RNA, and white indicates frozen RNA. (DOCX) [file pone.0111827.s004.docx]
