# Supplementary material for: Long Term Storage of Dry versus Frozen RNA for Next Generation Molecular Studies
Source: PLoS One. 2014 Nov 7;9(11):e111827. doi: 10.1371/journal.pone.0111827 (PMC4224406; doi:10.1371/journal.pone.0111827)
Supplement: Table S1 — qPCR threshold cycle (Ct) values for five frozen and desiccated samples using Roche TBP primer/probe mixes after two, six, and 12 months of storage. (DOCX) [file pone.0111827.s005.docx]

| Storage | Sample | Month 2 | Month 6 | month 12 |
| --- | --- | --- | --- | --- |
|  | SQ 100 | 30.38 | 29.21 | 32.73 |
|  | SQ 106 | 30.46 | 31.07 | 33.96 |
| Desiccation | SQ 110 | 29.55 | 29.18 | 32.92 |
| (Room | SQ 113 | 30.08 | 30.28 | 33.82 |
| Temperature) | SQ 112 | n/a | 32.39 | 31.40 |
|  | Average | 30.12 | 30.43 | 32.97 |
|  |  |  |  |  |
|  |  |  |  |  |
|  | Sample | Month 2 | Month 6 | month 12 |
|  | SQ 100 | 29.35 | 30.17 | 31.91 |
| Frozen | SQ 106 | 30.22 | 30.82 | 31.76 |
| (-80^o^C) | SQ 110 | 29.18 | 28.46 | 30.46 |
|  | SQ 113 | 29.95 | 29.98 | 31.53 |
|  | SQ 112 | n/a | 33.16 | 32.00 |
|  | Average | 29.68 | 30.52 | 31.53 |
