# Supplementary material for: Long Term Storage of Dry versus Frozen RNA for Next Generation Molecular Studies
Source: PLoS One. 2014 Nov 7;9(11):e111827. doi: 10.1371/journal.pone.0111827 (PMC4224406; doi:10.1371/journal.pone.0111827)
Supplement: Supplementary file 3 [file pone.0111827.s001.docx]

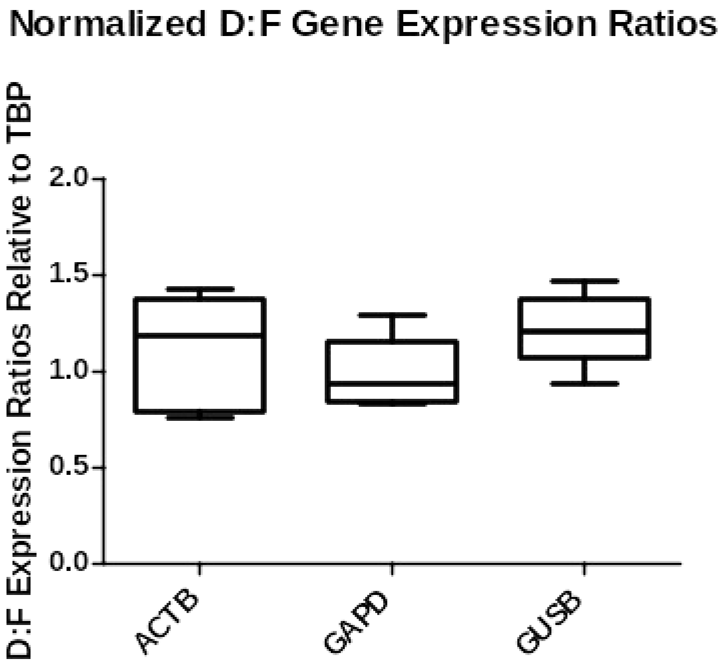
Normalized D:F Gene Expression Ratios
D:F Expression Ratios Relative to TBP
2.0
1.5
1.0
0.5
0.0
ACTB
GAPD
GUSB
