# Supplementary material for: Long Term Storage of Dry versus Frozen RNA for Next Generation Molecular Studies
Source: PLoS One. 2014 Nov 7;9(11):e111827. doi: 10.1371/journal.pone.0111827 (PMC4224406; doi:10.1371/journal.pone.0111827)
Supplement: Supplementary file 4 [file pone.0111827.s002.docx]

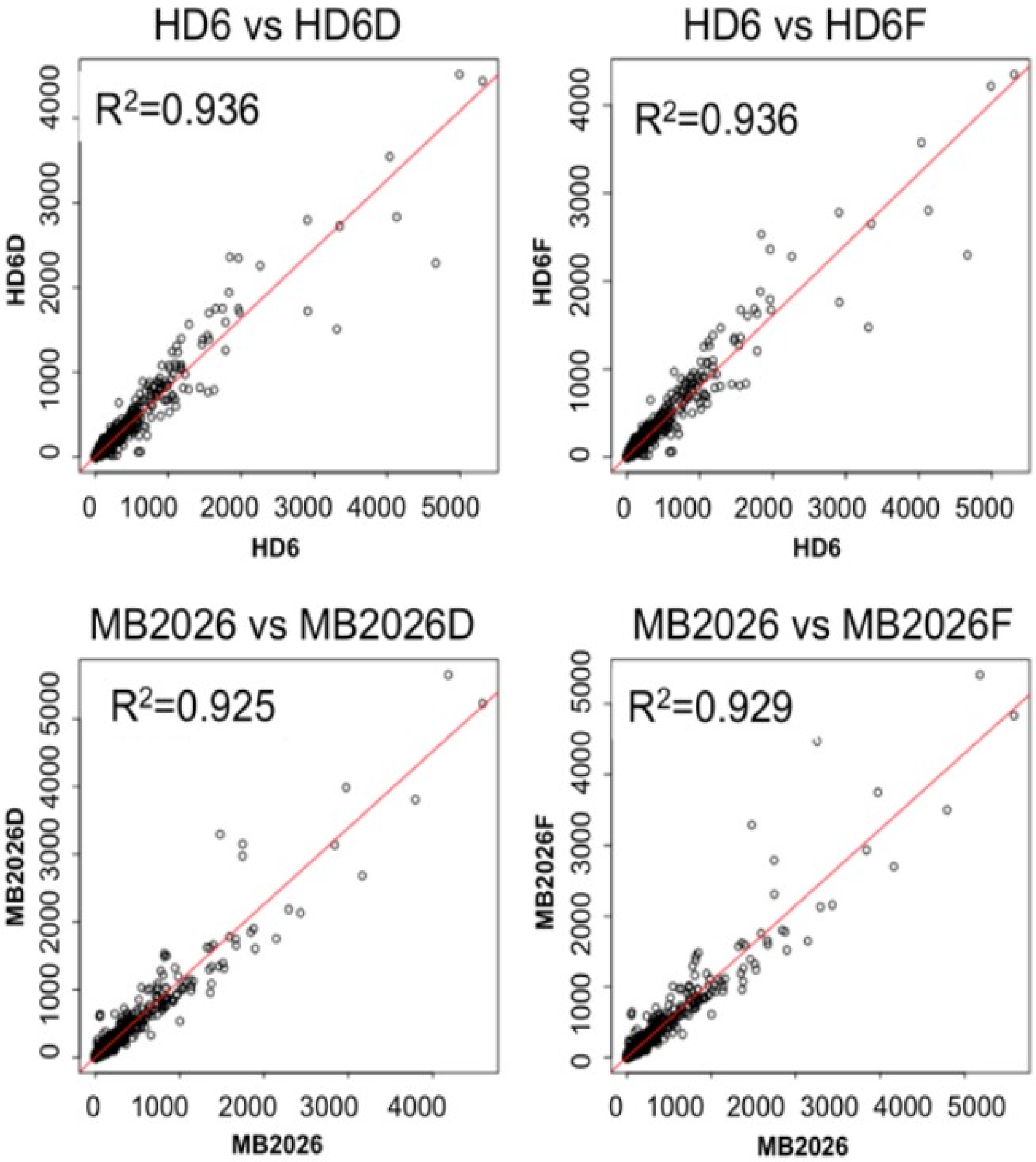

HD6 vs HD6D
R2=0.936
HD6D
HD6
HD6 vs HD6F
R2=0.936
HD6F
HD6
MB2026 vs MB2026D
R2=0.925
MB2026D
MB2026
MB2026 vs MB2026F
R2=0.929
MB2026F
MB2026
